# Investigation of the *ABCB1* Gene Polymorphism and Food Effects on the Avatrombopag Pharmacokinetics in Chinese Individuals: A Population Pharmacokinetic/Pharmacodynamic Analysis

**DOI:** 10.3390/ph18060903

**Published:** 2025-06-16

**Authors:** Xin Liu, Lulu Chen, Gehang Ju, Chao Li, Bijue Liu, Yunzhou Fei, Xintong Wang, Yang Gao, Qingfeng He, Xiao Zhu, Dongsheng Ouyang

**Affiliations:** 1 Department of Clinical Pharmacy and Pharmacy Administration, School of Pharmacy, Fudan University, Shanghai 200437, China; 2 Changsha Phamark Data Co., Ltd., Changsha 410013, China; 3 Hunan Key Laboratory for Bioanalysis of Complex Matrix Samples, Changsha Duxact Biotech Co., Ltd., Changsha 410013, China; chao.li@phamark.com (L.C.);; 4 Department of Pharmacy, The Affiliated Hospital, Xiangnan University, Chenzhou 423043, China; 5 Department of Clinical Pharmacology, Xiangya Hospital, Central South University, Changsha 410017, China; 6 Changsha Duxact Clinical Laboratory Co., Ltd., Changsha 410013, China

**Keywords:** avatrombopag, pharmacogenetics, pharmacokinetics, thrombocytopenia, personalized medicine

## Abstract

**Background/Objectives:** Avatrombopag (AVA), a thrombopoietin receptor agonist used to treat thrombocytopenia in patients with chronic liver disease, exhibits significant pharmacokinetic (PK) variability, particularly under fasting conditions. This study investigates the combined influence of food intake and genetic polymorphisms in *CYP2C9* and *ABCB1* on the PK and pharmacodynamics (PD) of AVA, with the goal of informing individualized dosing strategies. **Methods**: A pharmacogenetic analysis was conducted in 92 healthy participants, who received 20 mg of AVA under both fasting and fed conditions. A population PK/PD model was developed to evaluate the covariates effects on the PK variability. Monte Carlo simulations were used to predict AVA exposure and platelet count profiles under diverse dosing scenarios. **Results**: Food intake significantly reduced PK variability, with approximately 50% reductions in clearance (CL/F) and volume of distribution (Vd/F) compared to fasting conditions. Under fed conditions, *CYP2C9* intermediate metabolizers showed a 1.70-fold increase in exposure compared to normal metabolizers, but this difference was not observed under fasting conditions. *ABCB1* polymorphisms showed minimal impact, with the exception of *ABCB1* (*C1236T*) heterozygotes, which exhibited 1.37-fold increased exposure. Despite the observed PK variability, simulations demonstrated a consistent platelet count response across dosing regimens. **Conclusions**: While food intake and genetic polymorphisms in *CYP2C9* and *ABCB1* influenced AVA PK, these factors may not require dose adjustments, as platelet count responses remained consistent across genotypes and dosing conditions in the Chinese participants. These findings support simplified dosing strategies without the need for pharmacogenetic testing in Chinese individuals and may contribute to more individualized thrombocytopenia management.

## 1. Introduction

Thrombocytopenia is a hematologic disorder characterized by abnormally low platelet counts, often associated with inherited factors or drug-induced immunologic destruction [1]. Globally, the estimated incidence of thrombocytopenia in adults ranges from 1.6 to 3.9 cases per 100,000 person-years [2,3,4,5]. Despite the relatively low incidence of fatal bleeding events, which is approximately 0.13 per person-year, managing and preventing bleeding remains a significant challenge. Pharmacologic therapy is the primary approach due to its safety and convenience compared with the platelet transfusion [2,3]. Thrombopoietin receptor agonists (TPO-RAs) are commonly used as second-line treatments for immune thrombocytopenia (ITP). These agents mimic endogenous thrombopoietin to stimulate megakaryocyte proliferation and differentiation in the bone marrow [4,5,6].

Avatrombopag (AVA), a second-generation TPO-RA, is approved for the treatment of thrombocytopenia, particularly in patients with chronic liver disease (CLD) [7]. It is increasingly used in ITP patients due to its superior efficacy and favorable benefit–risk profile compared to other TPO-RAs [8]. Although AVA was widely used in clinical practice, the variability of its PK has raised concerns among clinicians. Previous studies have shown that food intake significantly impacts both the inter- and intra-individual variability of AVA exposure. Notably, between-subject variability (BSV) is up to two-fold higher in the fasting state compared to the fed state [9,10,11,12]. Thus, oral administration with food is recommended in clinical practice to reduce the variability and ensure consistent therapeutic effects.

Cytochrome P450 (*CYP*) *2C9* genetic polymorphisms were identified as other key contributors to AVA variability, as it is the primary metabolized enzyme [1,13] of AVA. The genetic polymorphisms significantly influence AVA exposure [1,9,10], with CYP2C9 *2, *3 alleles associated with higher exposure compared to *1 carriers. However, despite the AVA exposure caused by *CYP2C9* polymorphism, it had limited impact on the platelet count changes [1]. Beyond the metabolic factors, transporters also play a significant role in the AVA PK process. P-glycoprotein (P-gp) [7,14], encoded by the *ABCB1* gene, is primarily responsible for AVA disposition. Over 8000 single nucleotide polymorphisms (SNPs) [15] in *ABCB1* have been identified, influencing over 50% of the pharmacokinetic processes of various drugs [16]. Additionally, P-gp plays an important role in TPO-RAs transport, with polymorphisms impacting both drug exposure and efficacy [17,18]. However, the effect of P-gp polymorphisms on AVA remains underexplored. Therefore, this study focused on *CYP2C9* and *ABCB1* due to their primary roles in AVA metabolism and transport. Other genes, such as *CYP3A4,* were not included due to their limited contribution [9] and lack of consistent evidence in AVA disposition.

Therefore, this study aims to investigate the underlying causes of AVA PK variability, with a particular focus on the influence of *CYP2C9* and *ABCB1* gene polymorphisms. Given that the average lifespan of platelets in circulation is 7–10 days [19], a population pharmacokinetic-pharmacodynamic (PPK-PD) method was employed to evaluate whether differences in AVA exposure across subpopulations translate into meaningful changes in platelet count profiles under various dosing regimens.

## 2. Results

### 2.1. Demographic Characteristics

This analysis included 5923 AVA concentration data points from 92 healthy participants. Among these individuals, 83 were male and 9 were female. The median age was 28, with a range from 18 to 45. Four major gene polymorphisms were analyzed in this study; one in *CYP2C9* and three in *ABCB1*. For *CYP2C9* polymorphisms, 90% of participants were homozygous for the wild-type allele (*1/*1), while 10% were heterozygous (*1/*3). No individuals were identified as homozygous for the variant allele (*3/*3). The genotype frequencies for the three *ABCB1* variants were as follows:

For *ABCB1* (*C1236T*), 14% of participants were homozygous wild-type, 51% were heterozygous, and 35% were homozygous variant.

For *ABCB1* (*C3435T*), 40% were homozygous wild-type, 47% were heterozygous, and 13% were homozygous variant.

For *ABCB1* (*G2677T/A*), 20% were homozygous wild-type, 67% were heterozygous, and 13% were homozygous variant.

The details of the demographic information of the participants in this study are summarized in Table 1.

### 2.2. PK Assessment

#### 2.2.1. Food and Sex Effects on PK

The primary PK parameters of AVA under different food intake conditions and across sexes are summarized in Table 2. The C_max_ was significantly higher in the fed state compared to the fasting state, with a geometric least-squares mean ratio of 121.87% (95% CI: 105.45–140.85, *p* = 0.008), while AUC was minimally affected (Table S1). This suggests that food intake influences the PK profile of AVA in the Chinese population. Regarding sex differences, no statistically significant variation in AVA exposure was observed between males and females (Table S2).

#### 2.2.2. Genotypes Effects on PK

**Figure 1:**
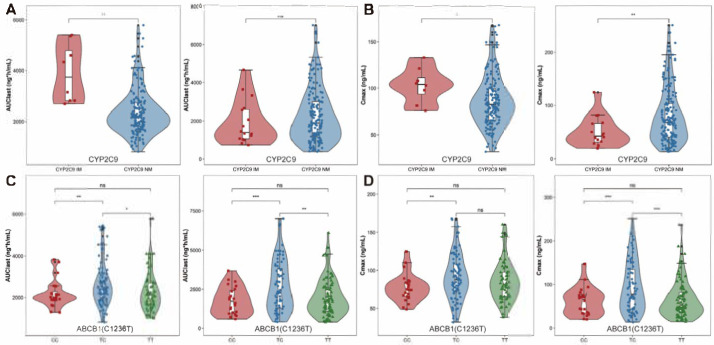
Comparison of the exposure parameters in different *CYP2C9* phenotypes and *ABCB1*(*C1236T*) genotypes in fasting (**left**) and fed state (**right**). (**A**): The AUC comparison between *CYP2C9* IM and NM phenotype; (**B**): The C_max_ comparison between *CYP2C9* IM and NM phenotype; (**C**): The AUC comparison between *ABCB1* (*C1236T*) CC, TC and TT genotype; (**D**): The C_max_ comparison between *ABCB1* (*C1236T*) CC, TC and TT genotype. Notes: ns, no significant difference; *, *p*-value < 0.05; **, *p*-value < 0.01; ***, *p*-value < 0.001. The colors represent distinct genotypes or phenotypes.

All genetic variants were in Hardy–Weinberg equilibrium. The PK parameters across different *CYP2C9* phenotypes and *ABCB1* genotypes are summarized in Table S3. For *CYP2C9* phenotypes, IMs exhibited significantly higher values for both t_1/2_ (25.68 vs. 17.72 h) and AUC_0-t_ (3704.04 vs. 2180.36 ng×h/mL) compared to NMs in the fed state, while C_max_ was not significantly affected by *CYP2C9* status (Table S4). In the fasting state, similar results were observed, with t_1/2_ substantially increased in the IM group relative to the NM group (*p* < 0.001). However, exposure parameters such as C_max_ and AUC_0-t_ displayed an unexpected trend, with the NM group showing slightly higher values than the IM group, though these differences were not statistically significant (Table S4; Figure 1A,B and Figure S1).

Regarding the effects of *ABCB1* genotypes on AVA pharmacokinetics, no significant differences were observed in C_max_ and AUC values across the *ABCB1* (*C3435T*) genotypes (Table S5, Figure S2A,B). Similarly, no significant associations were found between the *ABCB1* (*G2677T/A*) polymorphism and C_max_ or AUC parameters (Figure S2C,D). However, for *ABCB1* (*C1236T*), a significant difference in AVA exposure was detected between heterozygous and homozygous genotypes for both AUC and C_max_ (Figure 1C,D). In both fasting and fed states, individuals with homozygous wild-type and homozygous mutant genotypes displayed similar exposure levels, with C_max_ ratios of 102.43% in the fasting state and 113.67% in the fed state, and AUC_0-t_ ratios of 108.67% (fasting) and 108.38% (fed). In contrast, the exposure differences between heterozygous and homozygous genotypes were notable (Table S7, Figure 1). The C_max_ ratio of TT/TC was approximately 70.96% in the fasting state and 93.27% in the fed state. For TC/CC, the C_max_ and AUC_0-t_ ratios in the fasting state were 144.35% and 144.04%, respectively; similar trends were observed in the fed state, with C_max_ and AUC values of 121.87% and 120.93%, respectively. These findings suggest that the *ABCB1* (*C1236T*) polymorphism significantly influences AVA exposure. It warrants further investigation to explain its impact on drug response.

### 2.3. Population Pharmacokinetic Model of AVA

**Figure 2:**
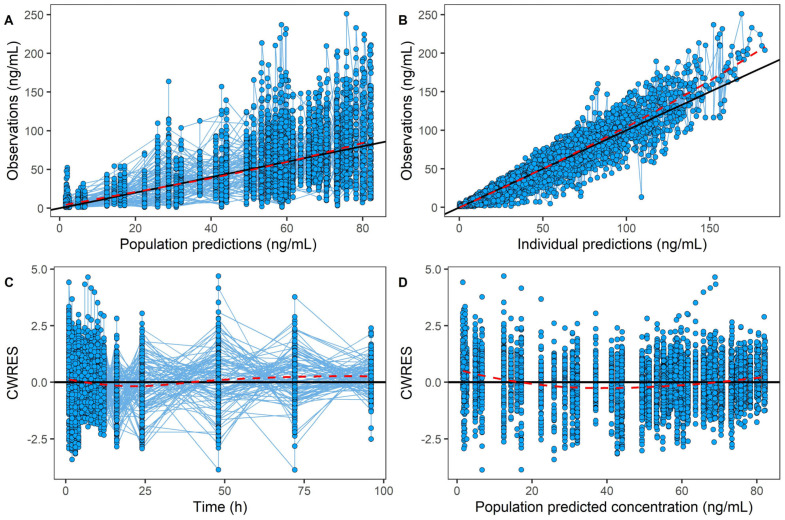
The goodness-of-fit plot for the final pharmacokinetic model of avatrombopag. Notes: CWRES, conditional weight residuals; the red dashed line indicates the linear regression trendline and the black solid line means the reference line. Blue circles represent observed data. (**A**) Observations vs. population predictions. (**B**) Observation vs. individual predictions. (**C**) Conditional weight residuals vs. time. (**D**) Conditional weight residuals vs. population predictions.

**Figure 3:**
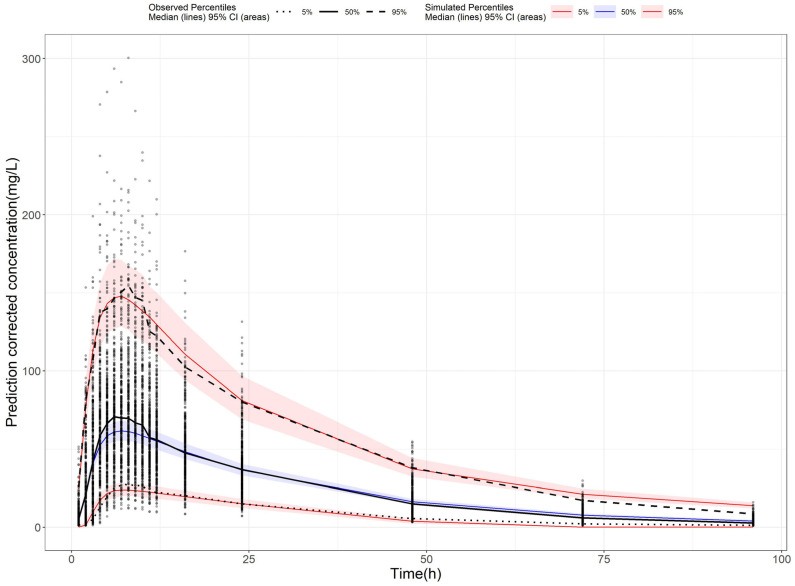
Visual predictive check plot of the final AVA population pharmacokinetic model. Notes: The circles represent observed data. Black lines represent the 5% (dashed), 50% (solid), and 95% (dashed) percentiles of the observed data. Shaded areas represent 95% confidence intervals of the median 5% (red), 50% (blue), and 95% (red) percentiles of the predicted concentrations.

To further investigate the effects of food intake and genetic polymorphisms on AVA exposure, a PPK model was developed. A one-compartment model with nine transit compartments, along with first-order absorption and elimination, was selected as the final model. The final model provided precise parameter estimates, with an apparent clearance (CL/F) of 7.85 L/h, an apparent volume of distribution (Vd/F) of 199 L, and an absorption constant (Ka) of 0.582/h. Most relative standard errors were below 30% (Table 3), demonstrating the model’s reliability. Incorporating the food–genotype co-effect on the absorption fraction (Fa) reduced the OFV by 15.96, indicating that both food and genotype co-effects significantly impact AVA absorption extent. The bootstrap analysis showed the parameter estimates from the final model closely matching the median values and falling within the 95% confidence interval (Table 3). This alignment confirms the stability and reproducibility of the model. Goodness-of-fit (GOF) diagnostic plots (Figure 2) showed unbiased model predictions and successful convergence. Additionally, the visual predictive check (VPC, Figure 3) demonstrated that most observed values were within the 95% prediction interval, confirming that the final model had strong predictive performance.

### 2.4. Population Pharmacokinetic and Pharmacodynamic Simulation

**Figure 4:**
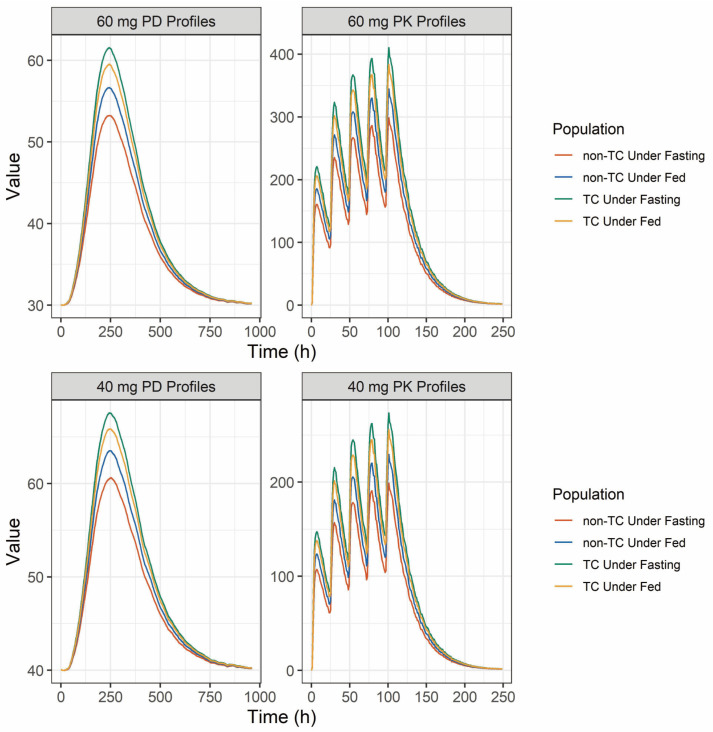
The PK/PD simulation profiles of AVA under 40 and 60 mg dosage regimen. Notes: the line represents the median value–time profiles of each *ABCB1 *(*C1236T*) genotype and food intake state.

Given that platelets have a short lifespan of 7–10 days in the circulation [19], it is important to investigate how different AVA exposure levels affect platelet counts in various groups with distinct exposure patterns. Simulation results for different AVA dosing regimens are summarized in Table 4 and illustrated in Figure 4. When dosed at 60 mg for five consecutive days, the maximum exposure difference between subpopulations was a 1.37-fold increase in C_max_ and AUC_τ_ (the comparison between the TC and non-TC under fasting state). Despite this, platelet counts showed no significant variation (61.56 × 10^9^ vs. 53.23 × 10^9^ for C_max_, 38,331.20 × 10^9^ vs. 35,907.80 × 10^9^ for AUC), indicating that dose adjustments based on *ABCB1* (*C1236T*) genotype or fasting state are unnecessary. Similar results were observed with a 40 mg dose for five consecutive days, where the PK exposure difference remained 1.37-fold, and platelet counts were nearly identical in these two subpopulations. Taken together, these results indicate that, although AVA exposure differed significantly by genotype and food condition, the resulting platelet count responses were only minimally affected.

## 3. Discussion

AVA, a thrombopoietin receptor agonist, is widely prescribed as a second-line treatment for thrombocytopenia in patients with CLD [20,21]. In this study, a single dose of 20mg AVA was administrated to healthy Chinese participants; a large variability was found in the fasting state. The pharmacogenetic analysis found a difference in C_max_ and AUC across *ABCB1* (*C1236T*) and *CYP2C9* genotypes in both the fasting and fed state. To further quantify the effects of the food and genotype, a PPK-PD model was developed. The results showed that, although a 1.37-fold exposure difference was observed between *ABCB1* (*C1236T*) homozygote and heterozygote in the fasting state, the resulting difference in platelet counts was minimal.

Food intake was found to significantly affect the variability of AVA’s PK parameters, especially C_max_, without altering the extent of absorption (Table S1). This aligns with findings from Nomoto et al. [1,9,10], who reported similar effects in a White population. The food intake influenced C_max_ but not mean PK values. The BSV for CL/F and Vd/F was notably higher in the fasting conditions compared to the fed states (62.4% vs. 36.6% for CL/F and 64.5% vs. 31.1% for Vd/F, respectively). Similar findings were also reported in the label of AVA, which directly supports the recommendation of taking medicine with meals. Since AVA is a Biopharmaceutical Classification System (BCS) Class II compound with low solubility [9], food may reduce PK variability by slowing gastrointestinal transit, thereby enhancing uniformity in AVA absorption and solubility. This mechanism explains the lower variability in the fed state [1].

AVA undergoes extensive metabolism primarily via *CYP2C9*, which accounts for approximately 44% of the administered dose [13]. Although in vitro studies suggest both *CYP2C9* and *CYP3A4* enzymes contribute equally, in vivo data indicate that *CYP2C9* plays a more dominant role [9]. Previous pharmacogenetic studies showed that *CYP3A* polymorphisms had no significant impact on AVA PK, while *CYP2C9* variants correlated with higher exposure in IMs than in NMs [1]. The allele frequency for CYP2C9*3 was 3.26%, while CYP2C9*1 accounted for 96.74% in this study, consistent with a previous report among Asian participants [22], which reported CYP2C9*3 had almost a 3% frequency in Asian population. *CYP2C9* IMs demonstrated higher exposure than NMs under fed conditions (Figure 2). However, in the fasting state, IMs exhibited lower exposure than NMs, likely due to high variability and the small sample size of IMs. Therefore, large numbers of clinical studies are needed to confirm these findings (Figure 1 and Table S4).

In addition to investigating *CYP2C9* polymorphism effects on the AVA exposure, this was the first study to evaluate the effects of *ABCB1* polymorphisms on AVA’s PK and PD characteristics. The *ABCB1* polymorphism is known to influence the bioavailability of oral drugs [23,24], potentially affecting clinical outcomes [25,26]. For *ABCB1* polymorphisms, three common variants were examined: *C1236T*, *G2677T/A*, and *C3435T*. The frequencies observed for each were similar to those reported in other Asian populations [27,28,29]. Specifically, for *ABCB1* (*C1236T*), 46.74% were heterozygous (TC), 40.22% were homozygous variant (TT), and 13.04% were homozygous wild-type (CC). When comparing single-dose PK parameters across *ABCB1* genotypes, the largest exposure difference was observed between the TC and CC genotypes in *ABCB1* (*C1236T*), with a 1.44-fold difference (Table S7). While there was no significant difference in exposure parameters detected between the different genotypes of *G2677T/A*, and *C3435T*.

Although our findings suggest a potential impact of the *C1236T* variant on AVA exposure, the underlying mechanisms remain unclear. This effect may be attributable to educed P-gp activity in TC carriers, resulting in decreased efflux and increased systemic concentrations. However, existing evidence on the functional impact of *ABCB1* polymorphisms is conflicting [26,30]. Studies have reported both increased [31,32,33] and decreased [34,35] ABCB1 mRNA or protein expression in relation to *3435T* or *2677T* alleles, depending on the population and tissue examined. The functional consequences of *C1236T* are even less consistent, with limited data suggesting possible effects on drug clearance for certain substrates [36]. Overall, the genotype–function relationship for ABCB1 remains poorly defined [16], and our findings warrant cautious interpretation and further mechanistic validation.

Given that AVA-induced platelet increases are evident 3–5 days after administration [1], with peak changes at 6–10 days, it is essential to assess how PK change translates into PD effects, especially in steady-state conditions. A PPK-PD model was developed to characterize the differences in PK and PD across genotypes. However, food intake had a substantial impact on the PK variability, and the effects of genetic polymorphisms differed between fasting and fed state. This indicated that food may play a more important role in AVA’s exposure and between-subjects variability. Thus, to better characterize the gene-food co-effects on AVA’s exposure, a more mechanistic model was developed. Although the PK data were obtained from healthy volunteers, the PD model was based on CLD patients, providing a clinically relevant PK-PD framework. We acknowledge that disease-related physiological changes may alter PK profiles; however, the primary objective of our analysis was not to predict absolute platelet counts in patients but to explore the relative influence of PK variability (e.g., due to genotype or food intake) on PD outcomes. This approach provides mechanistic insight into the robustness of platelet count outcomes despite inter-individual PK differences. The model not only considered the genotypes and food effects but also took the interaction between food and genotype into consideration. The results showed that, despite the significant exposure differences between *ABCB1* (*C1236T*) heterozygotes and homozygotes, platelet counts did not differ significantly between these groups, suggesting that dosage adjustments are not necessary based on *ABCB1* genotype alone.

This study has several limitations. First, the analysis population was derived from a bioequivalence study involving relatively homogeneous participants, which may limit the ability to identify certain covariates, such as body weight, that were identified in another study [10]. Second, the sample size for CYP2C9*3 carriers was quite low (n = 6), which may have affected the reliability of the results regarding *CYP2C9* IMs in the fasting state. While our study provides novel insights into the interplay between genotype, food intake, and AVA pharmacokinetics, the clinical implications of these findings require further validation. Future work should incorporate larger, more genetically diverse patient cohorts to assess whether PK variability may translate into PD alterations in real-world settings.

## 4. Materials and Methods

### 4.1. Study Population

This study included healthy volunteers enrolled from a single-center, two-sequence, four-period bioequivalence trial conducted at the Clinical Trials Unit of Hunan Province People’s Hospital (Changsha, Hunan). All trials were performed in compliance with the principles outlined in the Declaration of Helsinki for biomedical research involving human subjects. Informed consent was obtained from all participants for both clinical trial participation and pharmacogenetic analysis. Participants were free to withdraw from the study at any time. This study was approved by the Institutional Review Board (IRB) in the Hunan Province People’s Hospital (approval number: [2023]-32.1).

### 4.2. Study Design and Pharmacokinetic Assessments

Participants received a single 20 mg dose of AVA with 200 mL of water under both fasting and fed conditions. In the fed condition, subjects consumed a high-fat, high-calorie meal approximately 30 min prior to dosing, providing a total of 800–1000 kcal, approximately 150 kcal from protein, 250 kcal from carbohydrates, and 500–600 kcal from fat. This open-label, crossover study consisted of four periods with two sequences, separated by a 14-day washout. Blood samples were collected at pre-specified times: pre-dose (0 h) and at 1, 2, 3, 4, 5, 6, 7, 8, 9, 10, 11, 12, 16, 24, 48, 72, and 96 h post-dose. Samples were centrifuged at 3500 rpm for 10 min at 4 °C and stored at −70 °C until analysis.

AVA concentrations were quantified using ultra-performance liquid chromatography-tandem mass spectrometry (UPLC-MS/MS) with an AVA-D8 internal standard (UPLC I-Class and Xevo TQ-S, Waters Corporation, Milford, MA, USA). The analysis was performed on a Waters ACQUITY UPLC-BEH C18 column (1.7 μm, 2.1 mm × 50 mm) with a mobile phase of 2 mM ammonium acetate (0.1% formic acid) and acetonitrile as phase A and B, respectively, using isocratic elution at a flow rate of 0.4 mL/min. Detection employed an electrospray ionization source (ESI) in positive ion mode. The ion pairs monitored were m/z 649.44→266.95 for AVA and m/z 659.10→267.00 for AVA-D8, with a linear range of 1.000–400.000 ng/mL. The retention time for both the analyte and internal standard was 0.63 ± 0.1 min. Following current US FDA bioanalytical validation guidelines, this modified method for AVA quantification in plasma was validated and deemed reliable for pharmacokinetic studies.

### 4.3. Genotyping of CYP2C9 and ABCB1

*CYP2C9* and *ABCB1* DNA was extracted from blood samples using the Universal Genomic DNA Extraction Kit provided by GenMagBio (Beijing, China) and quantified using an Ultra-micro spectrophotometer-Nano Drop One provided by Thermofisher (Waltham, MA, USA). Genotyping was performed by Duxact Inc. (Changsha, China), focusing on CYP2C9*1, *3 (rs1057910), *ABCB1* (*C1236T*) (rs1128503), *ABCB1* (*G2677T/A*) (rs2032582), and *ABCB1* (*C3435T*) (rs1045642) variants. The TaqMan MGB Probe Method on a qPCR platform was used to identify SNPs. Volunteers were grouped based on their genotype; samples without a clear amplification profile were excluded from analysis. For *CYP2C9*, the *1/*1 carriers were classified into the normal metabolizer (NM) group, the individuals carrying the *1/*3 variant were classified into the intermediate metabolizer (IM) group, and the *3/*3 were assigned to the poor metabolizer (PM) group.

### 4.4. Population Pharmacokinetic Model

Non-linear mixed-effects modeling was conducted using NONMEM (version 7.5, ICON Development Solutions, San Antonio, TX, USA) with the first-order conditional estimation with interaction (FOCE-I) algorithm. Inter-individual variability (IIV) was modeled using an exponential approach, while residual variability was tested using proportional, additive, and combined error models, and the best performing one was selected. Covariate analysis included demographic data (e.g., age, weight, sex), lab test results (e.g., ALT, AST), clinical trial conditions (e.g., food intake), and genotype information. A stepwise covariate modeling strategy was used (forward selection: *p* < 0.05; backward elimination: *p* < 0.01). Covariates were selected based on statistical significance and clinical relevance.

Model evaluation was performed using successful convergence, objective function value (OFV), Akaike information criterion (AIC), parameter precision, goodness-of-fit (GOF) plots, visual predictive checks (VPCs), and non-parametric bootstrap analysis to confirm model robustness.

### 4.5. Simulations of PPK-PD

To investigate the potential impact of PK variability on the PD response of AVA, a previously published PD model developed in chronic liver disease (CLD) patients [10] was adopted and linked to our PK model derived from healthy Chinese participants. Ethnic sensitivity was accounted for by applying a 32% slope reduction for East Asians. This enabled simulation of platelet count trajectories under different PK profiles, stratified by food intake and *ABCB1* genotype. Simulations were performed using a typical virtual patient, in which key covariates (e.g., genotype, food status) were varied to assess their impact.

Model simulations were conducted under two dosing scenarios (60 mg QD for platelet count <40 × 10^9^/L; 40 mg QD for 40–50 × 10^9^/L; both for 5 consecutive days) [7] to evaluate the impact of the genotypes (TC vs. non-TC) and food conditions on drug exposure and platelet count profiles. The key exposure parameters (PK: C_max_, AUC_τ_ in the last dose; PD: platelet C_max_, AUC_0-t_ from day 1 to day 40) were compared to reveal the exposure and response difference in different populations.

### 4.6. Statistical Analysis

Non-compartmental analysis was conducted using WinNonLin (Version 6.3, Certara, Pennsylvania, PA, USA) to calculate PK parameters, including terminal half-life (t_1/2_), maximum plasma concentration (C_max_), time to reach C_max_ (t_max_), area under the plasma concentration–time curve up to the last measurable concentration (AUC_0–t_), and AUC extrapolated to infinity (AUC_0–∞_). ANOVA, following natural log-transformation of major PK parameters, was used to evaluate the effects of food intake, sex, and genotype on AVA pharmacokinetics.

## 5. Conclusions

This study provides important insights into the PK and PD of AVA, particularly in relation to food intake and genetic variability in *CYP2C9* and *ABCB1*. Our findings confirmed that food significantly reduces PK variability. Additionally, although *CYP2C9* or *ABCB1* gene polymorphism had statistically significant effects on AVA’s exposure, they had no clinically significant impact on AVA’s PD response in the Chinese participants. There was no need to adjust the doses according to the genotypes of *CYP2C9* or *ABCB1* for the Chinese participants.

## Figures and Tables

**Table 1:** Demographic characteristics of this study.

Characteristic	Fasting (n = 47)	Fed (n = 45)
	Mean ± SD	Mean ± SD
Age (years)	28.98 ± 6.27	28.48 ± 7.55
Body weight (kg)	63.22 ± 7.88	60.95 ± 6.32
Height (cm)	167.62 ± 6.82	167.16 ± 5.67
Sex (Male/Female)	41/6	42/3
Alanine Aminotransferase (U/L)	23.04 ± 12.35	19.46 ± 12.31
Aspartate Aminotransferase (U/L)	21.2 ± 4.71	18.97 ± 4.5
Albumin (g/L)	47.06 ± 2.25	47.19 ± 2.07
Total Protein (g/L)	74.6 ± 4.18	74.26 ± 3.54
Total Bilirubin (µmol/L)	14.76 ± 4.42	14.38 ± 6.92
Direct Bilirubin (µmol/L)	3.89 ± 1.27	3.63 ± 1.77
Prothrombin Time (s)	13.65 ± 0.73	12.4 ± 0.74
Activated Partial Thromboplastin Time (s)	30.36 ± 3.35	35.8 ± 3.39
International Normalized Ratio	1.12 ± 0.07	1.03 ± 0.06
Fasting Blood Glucose (mmol/L)	2.7 ± 0.49	2.41 ± 0.34
Creatinine (µmol/L)	76.26 ± 11.03	76.79 ± 10.56
Blood Urea Nitrogen (mmol/L)	4.06 ± 0.69	4.33 ± 0.83
Uric Acid (µmol/L)	387.44 ± 79.85	360.76 ± 76.24
*CYP2C9* (NM/IM)	43/4	43/2
*ABCB1* (*C1236T*) (CC/TC/TT)	7/28/12	6/19/20
*ABCB1* (*C3435T*) (CC/TC/TT)	22/19/6	15/24/6
*ABCB1* (*G2677T/A*) (GA/GG/TA/TG/TT)	3/9/4/23/8	10/9/5/17/4

**Table 2:** Summary statistics for the main pharmacokinetic parameters of males and females under fasting and fed conditions.

PK Parameters	Fasting			Fed		
	ALL (N = 47)	Male (N = 41)	Female (N = 6)	ALL (N = 45)	Male (N = 42)	Female (N = 3)
t_1/2_ (h)	18.92 ± 3.56	19.27 ± 3.60	16.55 ± 2.09	18.18 ± 2.91	18.14 ± 3.02	19.51 ± 1.69
t_max_ (h)	7.02(3.00~16.00)	7.02(3.00~10.02)	6.51(4.00~16.00)	6.00(2.00~12.00)	5.00(2.00~12.00)	5.01(4.00~10.02)
C_max_ (ng/mL)	80.44 ± 52.70	77.22 ± 49.15	101.90 ± 71.02	85.06 ± 27.99	86.00 ± 28.51	73.92 ± 25.77
AUC_0-t_ (h×ng/mL)	2335.67 ± 1456.90	2302.61 ± 1430.75	2556.05 ± 1672.36	2389.39 ± 933.23	2415.02 ± 970.48	2018.66 ± 615.38
AUC_0-∞_ (h×ng/mL)	2428.42 ± 1502.16	2399.72 ± 1479.64	2619.79 ± 1702.32	2478.66 ± 1014.60	2506.96 ± 1058.14	2095.01 ± 625.05

Note: tmax expressed as median for range. Other values are presented as mean ± standard deviation.

**Table 3:** The final parameters estimate of avatrombopag and bootstrap results.

Parameters	Final Model		Bootstrap
	Estimates	RSE%	Median [2.5th–97.5th]
CL/F (L/h)	7.85	14%	7.81 [5.92–10.62]
Vd/F (L)	199	13%	198.01 [149.77–267.64]
ka (/h)	0.582	13%	0.581 [0.4723–0.785]
MTT (h)	1.33	5%	1.33 [1.22–1.45]
COV1 *	−0.0653	111%	−0.0702 [−0.3069–0.2698]
COV2 *	−0.272	15%	−0.28 [−0.47–0.01]
COV3 *	0.177	56%	0.186 [−0.216–0.431]
**Between-subject variability**			
CL/F	0.182	35%	0.174 [0.126–0.235]
Vd/F	0.159	32%	0.153 [0.106–0.204]
KA	0.504	35%	0.48 [0.29–0.78]
MTT	0.131	23%	0.128 [0.093–0.169]
OCC [KA]	0.913	12%	0.922 [0.681–1.346]
**Residual unexplained variability**			
σ_prop_ (%)	23.90%	4%	23.79% [22.28–25.74%]
σ_add_ (ng/mL)	1.18	3.00%	1.17 [0.86–1.48]

Notes: CL/F: the apparent plasma clearance; Vd/F: the apparent volume distribution; Ka: the absorption rate constant; MTT: the mean transit time; RSE: relative standard error; OCC: occasional variability; σ_prop_: proportional error; σ_add_: additional error; COV1: the effect of fed state and TC genotype on Fa; COV2: the effect of fast state and non-TC genotype on Fa; COV3: the effect of fed state and non-TC genotype on Fa. * Fa = 1 × (1 + COV1 × FOOD + COV2 × *ABCB1* + COV3 × FOOD × *ABCB1*). If in the fed state, FOOD is 1; fasting state is 0. If the genotype is heterozygote, *ABCB1* is 0; homozygote is 1.

**Table 4:** The simulated PK/PD results of different *ABCB1 *(*C1236T*) genotypes under different food intake states.

Dosage Regimen	Group	PK		PD	
		C_max_ (ng/mL)	AUC (ng × h/mL)	C_max_ (×10^9^)	AUC (×10^9^)
60 mg	Non-TC under Fasting State	160.92	5480.21	53.23	35,907.80
	Non-TC under Fed State	185.54	6321.18	56.67	36,911.66
	TC under Fasting State	220.88	7527.41	61.56	38,331.20
	TC under Fed State	206.50	7035.84	59.55	37,750.24
40 mg	Non-TC under Fasting State	107.24	3653.12	60.64	44,904.43
	Non-TC under Fed State	123.76	4214.01	63.51	45,807.92
	TC under Fasting State	147.41	5018.61	67.58	47,086.80
	TC under Fed State	137.80	4691.04	65.85	46,569.38

Note: Simulated using typical individual parameters. For PK, AUC represents the 24 h area under the curve after the last dose, and C_max_ represents the maximum concentration after the last dose. For PD, AUC represents the area under the curve from 0 to 960 h, and C_max_ means the maximum platelet count from 0 to 960 h.

## Data Availability

The data that support the findings of this study are available from the corresponding author upon reasonable request.

## Supplementary Materials

The following supporting information can be downloaded at: https://www.mdpi.com/article/10.3390/ph18060903/s1.
