# Supplementary material for: Investigation of the ABCB1 Gene Polymorphism and Food Effects on the Avatrombopag Pharmacokinetics in Chinese Individuals: A Population Pharmacokinetic/Pharmacodynamic Analysis
Source: Pharmaceuticals (Basel). 2025 Jun 16;18(6):903. doi: 10.3390/ph18060903 (PMC12196290; doi:10.3390/ph18060903)
Supplement: Supplementary file 1 [file pharmaceuticals-18-00903-s001.zip › pharmaceuticals-3641562-supplementary.pdf]

Table S1 Comparison of pharmacokinetic parameters under fasting and fed.

PK parameter	Fed	Fasting	Fed/Fasting ratio	Fed/Fasting 95%CI	pValue
C_{max}(ng/mL)	80.74	66.25	121.87	105.45-140.85	0.008
AUC_{0-t}(h*ng/mL)	2240.5	1952.13	114.77	99.46-132.44	0.059
$AUC_{0-\infty}$(h*ng/mL)	2314.98	2037.02	113.65	98.53-131.08	0.079

Notes: The ANOVA was used in analysis. CI Confidence Interval. C_{max}, maximum plasma concentration; AUC_{0-t}, area under the plasma concentration-time curve from the time of administration up to the last time point with a measurable concentration post-dose; $AUC_{0-\infty}$, AUC extrapolated to infinity.

Table S2 Comparison of pharmacokinetic parameters between Female and Male subjects under fasting and fed state

Group	PK parameter	Female	Male	Female/Male ratio	Female/Male 95%CI	pValue
Fasting	C_{max}(ng/mL)	78.02	64.65	120.69	82.08-177.45	0.335
	AUC_{0-t}(h*ng/mL)	2065.68	1935.65	106.72	73.47-155.02	0.73
	$AUC_{0-\infty}$(h*ng/mL)	2127.23	2023.82	105.11	72.69-151.98	0.789
Fed	C_{max}(ng/mL)	69.62	81.63	85.28	64.66-112.49	0.256
	AUC_{0-t}(h*ng/mL)	1931.56	2257.39	85.57	63.32-115.62	0.306
	$AUC_{0-\infty}$(h*ng/mL)	2008.71	2332.42	86.12	63.27-117.22	0.338

Notes: The ANOVA was used in analysis. CI Confidence Interval. C_{max}, maximum plasma concentration; AUC_{0-t}, area under the plasma concentration-time curve from the time of administration up to the last time point with a measurable concentration post-dose; $AUC_{0-\infty}$, AUC extrapolated to infinity.

Table S3 Comparison of pharmacokinetic parameters between different genotypes

Gene (Variant)	Genotype/phenotype	Fasting			Fed		
		AUC_{0-t}(ng*h/mL)	C_{max} (ng/mL)	$t_{1/2}$ (h)	AUC_{0-t}(ng*h/mL)	C_{max} (ng/mL)	$t_{1/2}$ (h)
CYP2C9	IM	1921.96±939.06	51.06±19.13	24.30±4.15	3877.63±1342.09	99.46±26.34	25.87±3.63
	NM	2375.07±1494.89	83.24±54.06	18.40±3.06	2317.06±880.56	84.49±28.42	17.87±2.40
ABCB1 (C3435T)	C/C	2613.59±1465.06	88.34±50.41	19.57±4.34	2323.56±914.21	85.14±27.61	17.95±3.25
	C/T	2273.82±1541.65	81.00±57.39	18.39±3.20	2551.40±1060.53	87.05±29.92	18.96±2.34
	T/T	2110.41±1023.15	64.06±29.82	20.05±3.30	2093.54±648.05	79.50±27.42	16.94±3.30
ABCB1 (C1236T)	C/C	1844.75±949.84	64.76±34.60	20.16±4.83	2038.51±620.58	71.32±16.14	17.10±0.93
	C/T	2823.73±1717.04	99.07±60.77	18.30±2.86	2542.12±984.49	89.74±29.28	18.63±3.19
	T/T	2052.71±1214.42	68.79±44.98	19.09±3.67	2291.46±989.08	83.72±29.48	18.08±3.05
ABCB1 (G2677T/A)	T/T	2049.73±991.37	61.66±30.21	19.89±3.10	2234.54±692.09	86.06±31.04	17.52±3.44
	G/C/T/A	2479.71±1620.5	87.52±59.17	18.71±3.76	2367.4±935.43	82.96±27.54	18.32±2.32
	G/G	2079.59±1142.14	72.04±38.62	18.80±3.22	2527.11±1131.55	92.40±30.39	18.25±4.51

Notes: Values are presented as mean ± standard deviation. IM, the intermediate metabolizers. NM, the normal metabolizers.

Table S4 Comparison of the statistical analysis of pharmacokinetic parameters of different type of *CYP2C9* metabolizers

Group	PK parameter	IM	NM	IM/NM ratio	IM/NM 95%CI	pValue
Fasting	$t_{1/2}$(h)	23.97	18.17	131.89	117.34-148.23	<0.001
	C_{max}(ng/mL)	47.96	68.32	70.2	44.44-110.89	0.128
	AUC_{0-t}(h*ng/mL)	1741.46	1973.48	88.24	56.50-137.81	0.579
	$AUC_{0-\infty}$(h*ng/mL)	1883.58	2052.27	91.78	59.08-142.59	0.7
Fed	$t_{1/2}$(h)	25.68	17.72	144.94	126.63-165.90	<0.001
	C_{max}(ng/mL)	96.94	80.05	121.1	86.62-169.30	0.259
	AUC_{0-t}(h*ng/mL)	3704.04	2180.36	169.88	119.90-240.70	0.003
	$AUC_{0-\infty}$(h*ng/mL)	4044.19	2247.96	179.9	126.35-256.15	0.001

Notes: The ANOVA was used in analysis. CI Confidence Interval. C_{max}, maximum plasma concentration; AUC_{0-t}, area under the plasma concentration-time curve from the time of administrationup to the last time point with a measurable concentration post-dose; $AUC_{0-\infty}$, AUC extrapolated to infinity. IM, the intermediate metabolizers. NM, the normal metabolizers.

Table S5 Comparison of pharmacokinetic parameters of different type of metabolizers-*ABCB1*(G3435T)

Group	PK parameter	TT	TC	CC	TT/CC ratio	TT/CC pValue	TT/CC 95%CI	TT/TC ratio	TT/TC pValue	TT/TC 95%CI	TC/CC ratio	TC/CC pValue	TC/CC 95%CI
Fasting	C_{max}(ng/mL)	55.99	65.51	74.88	74.77	0.185	48.52-115.22	85.46	0.418	58.23-125.42	87.49	0.392	64.26-119.13
	AUC_{0-t}(h*ng/mL)	1824.89	1878.89	2225.85	81.99	0.347	53.99-124.50	97.13	0.876	67.04-140.71	84.41	0.262	62.65-113.74
	$AUC_{0-\infty}$(h*ng/mL)	1927.6	1954.54	2324.1	82.94	0.37	54.91-125.28	98.62	0.94	68.40-142.20	84.1	0.246	62.65-112.88
Fed	C_{max}(ng/mL)	76.39	82.51	80.49	94.9	0.635	76.28-118.07	92.58	0.488	74.31-115.34	102.5	0.747	88.06-119.31
	AUC_{0-t}(h*ng/mL)	2010.13	2377.22	2174.61	92.44	0.507	73.11-116.87	84.56	0.161	66.78-107.07	109.32	0.281	92.87-128.68
	$AUC_{0-\infty}$(h*ng/mL)	2070.31	2463.81	2244.77	92.23	0.504	72.55-117.24	84.03	0.156	66.00-106.98	109.76	0.27	92.89-129.68

Notes: The ANOVA was used in analysis. CI Confidence Interval. C_{max}, maximum plasma concentration; AUC_{0-t}, area under the plasma concentration-time curve from the time of administration up to the last time point with a measurable concentration post-dose; $AUC_{0-\infty}$, AUC extrapolated to infinity.

Table S6 Comparison of pharmacokinetic parameters of different type of metabolizers-*ABCB1*(G2677T/A)

Group	PK parameter	TT	G/A/T	GG	TT/GG ratio	TT/GG pValue	TT/GG 95%CI	TT/GAT ratio	TT/GAT pValue	TT/GAT 95%CI	GAT/GG ratio	GAT/GG pValue	GAT/GG 95%CI
Fasting	C_{max}(ng/mL)	52.93	70.88	63.63	83.2	0.684	49.1-141	74.7	0.242	48.6-115	111	0.804	74.0-168
	AUC_{0-t}(h*ng/mL)	1781.48	2037.43	1819.75	97.9	0.995	58.6-164	87.4	0.725	57.5-133	112	0.778	75.2-167
	$AUC_{0-\infty}$(h*ng/mL)	1879.02	2122.3	1892.63	99.3	0.999	59.8-165	88.5	0.763	58.6-134	112	0.767	75.7-166
Fed	C_{max}(ng/mL)	82.5	78.75	87.23	94.6	0.917	67.6-132	105	0.926	77.9-141	90.3	0.484	73.1-112
	AUC_{0-t}(h*ng/mL)	2146.82	2219.89	2321.61	92.5	0.867	64.1-133	96.7	0.967	69.9-134	95.6	0.889	75.9-120
	$AUC_{0-\infty}$(h*ng/mL)	2211.8	2294.19	2405.15	92	0.855	63.2-134	96.4	0.963	69.2-134	95.4	0.882	75.3-121

Notes: The ANOVA was used in analysis. CI Confidence Interval. C_{max}, maximum plasma concentration; AUC_{0-t}, area under the plasma concentration-time curve from the time of administration up to the last time point with a measurable concentration post-dose; $AUC_{0-\infty}$, AUC extrapolated to infinity.

Table S7 Comparison of pharmacokinetic parameters of different type of metabolizers-*ABCB1*(C1236T)

Group	PK parameter	TT	TC	CC	TT/CC ratio	TT/CC pValue	TT/CC 95%CI	TT/TC ratio	TT/TC pValue	TT/TC 95%CI	TC/CC ratio	TC/CC pValue	TC/CC 95%CI
Fasting	C_{max}(ng/mL)	57.89	81.59	56.52	102.43	0.904	68.99-152.08	70.96	0.014	54.09-93.09	144.35	0.073	96.55-215.82
	AUC_{0-t}(h*ng/mL)	1762.01	2335.48	1621.37	108.67	0.668	74.03-159.52	75.45	0.036	57.96-98.20	144.04	0.067	97.47-212.87
	$AUC_{0-\infty}$(h*ng/mL)	1844.22	2420.7	1708.62	107.94	0.69	73.84-157.78	76.19	0.041	58.70-98.88	141.68	0.077	96.28-208.48
Fed	C_{max}(ng/mL)	79.24	84.96	69.71	113.67	0.254	91.04-141.92	93.27	0.366	80.08-108.63	121.87	0.065	98.72-150.45
	AUC_{0-t}(h*ng/mL)	2132.49	2379.4	1967.52	108.38	0.509	85.16-137.95	89.62	0.192	75.94-105.78	120.93	0.102	96.19-152.04
	$AUC_{0-\infty}$(h*ng/mL)	2201.64	2467.64	2014.59	109.28	0.476	85.41-139.83	89.22	0.184	75.32-105.68	122.49	0.088	96.94-154.77

Notes: The ANOVA was used in analysis. CI Confidence Interval. C_{max}, maximum plasma concentration; AUC_{0-t}, area under the plasma concentration-time curve from the time of administration up to the last time point with a measurable concentration post-dose; $AUC_{0-\infty}$, AUC extrapolated to infinity.

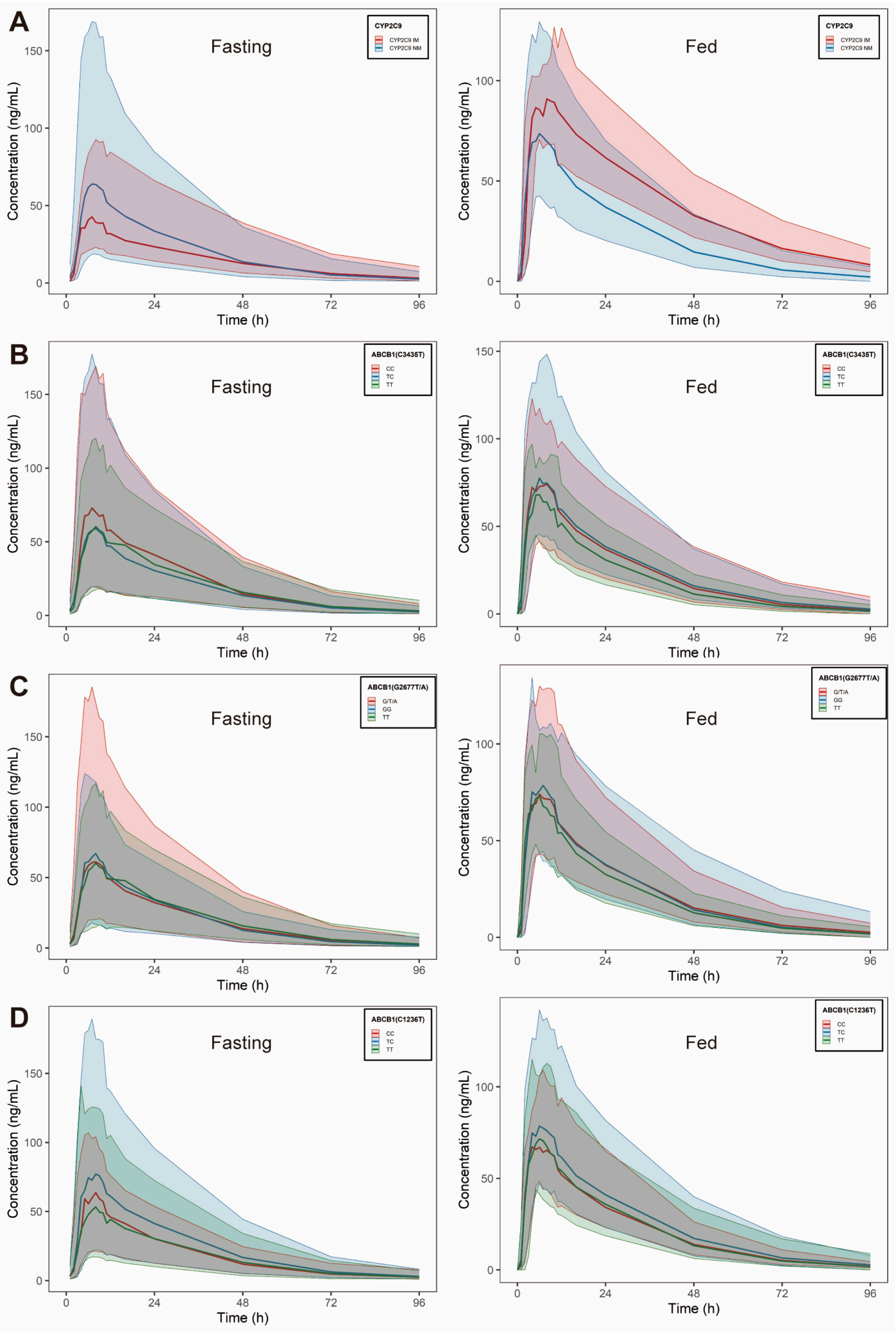

Figure S1 Concentration–time profiles of avatrombopag across different genotypes under fasting and fed conditions.

Notes: (A) the concentration-time profiles in *CYP2C9* NM and IM; (B) the concentration-time profiles in different *ABCB1*(C3435T) genotypes; (C) the concentration-time profiles in different *ABCB1*(G2677T/A) genotypes; (D) the concentration-time profiles in different *ABCB1*(C1236T) genotypes. NM, normal metabolizers; IM, intermediate metabolizers.

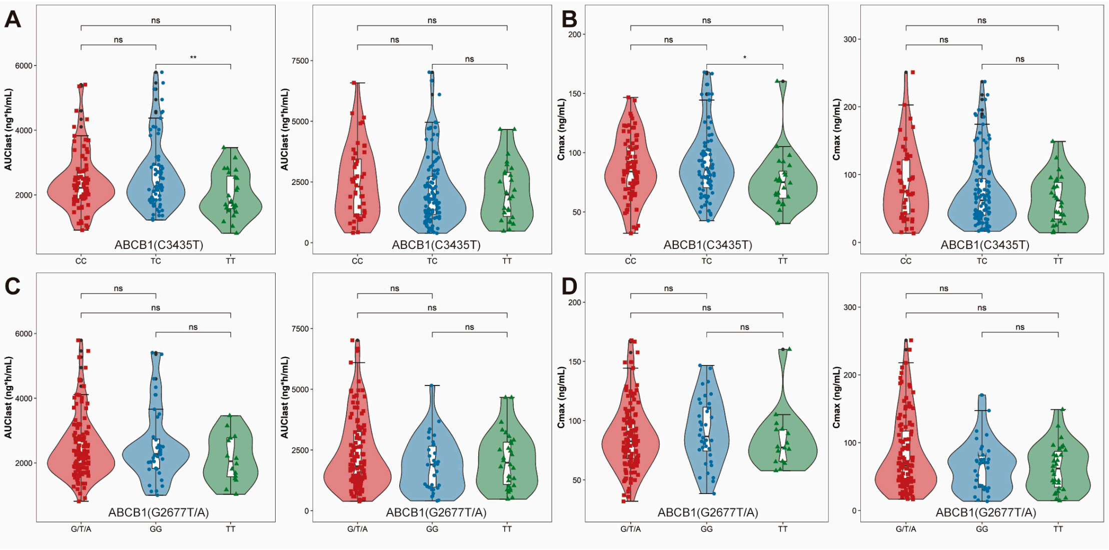

Figure S2 Comparison of the exposure parameters in different *ABCB1(C3435T)* and *(G2677T/A)* genotypes in fasting (left) and fed state (right).

Notes: ns, no significant difference; *, represents the p-value <0.05; **, represents the p-value <0.01.
